# Salivary α-amylase exhibits antiproliferative effects in primary cell cultures of rat mammary epithelial cells and human breast cancer cells

**DOI:** 10.1186/1756-9966-30-102

**Published:** 2011-10-25

**Authors:** Maren Fedrowitz, Ralf Hass, Catharina Bertram, Wolfgang Löscher

**Affiliations:** 1 Department of Pharmacology, Toxicology, and Pharmacy, University of Veterinary Medicine, Buenteweg 17, Hannover, 30559, Germany; 2 Biochemistry and Tumor Biology Lab, Gynecology Research Unit, Department of Obstetrics and Gynecology, Carl-Neuberg-Str. 1, Medical University, Hannover, 30625, Germany

**Keywords:** amylase, cell proliferation, breast cancer, primary cell culture, mammary gland

## Abstract

**Background:**

Breast cancer is one of the most diagnosed cancers in females, frequently with fatal outcome, so that new strategies for modulating cell proliferation in the mammary tissue are urgently needed. There is some, as yet inconclusive evidence that α-amylase may constitute a novel candidate for affecting cellular growth.

**Methods:**

The present investigation aimed to examine if salivary α-amylase, an enzyme well known for the metabolism of starch and recently introduced as a stress marker, is able to exert antiproliferative effects on the growth of mammary gland epithelial cells.

For this purpose, primary epithelial cultures of breast tissue from two different inbred rat strains, Fischer 344 (F344) and Lewis, as well as breast tumor cells of human origin were used. Treatment with human salivary α-amylase was performed once daily for 2 days followed by cell counting (trypan blue assay) to determine alterations in cell numbers. Cell senescence after α-amylase treatment was assessed by β-galactosidase assay. Endogenous α-amylase was detected in cells from F344 and Lewis by immunofluorescence.

**Results:**

Salivary α-amylase treatment in vitro significantly decreased the proliferation of primary cells from F344 and Lewis rats in a concentration-dependent manner. Noticeably, the sensitivity towards α-amylase was significantly higher in Lewis cells with stronger impact on cell growth after 5 and 50 U/ml compared to F344 cells. An antiproliferative effect of α-amylase was also determined in mammary tumor cells of human origin, but this effect varied depending on the donor, age, and type of the cells.

**Conclusions:**

The results presented here indicate for the first time that salivary α-amylase affects cell growth in rat mammary epithelial cells and in breast tumor cells of human origin. Thus, α-amylase may be considered a novel, promising target for balancing cellular growth, which may provide an interesting tool for tumor prophylaxis and treatment.

## Background

In females, breast cancer still ranks among the primary reasons of death caused by cancer [1]. Thus, new approaches for regulating cell proliferation in the mammary gland are required for the development of improved therapies. Numerous factors and molecular pathways have already been reported to influence proliferation and carcinogenesis in the mammary gland [2,3], and new findings are constantly provided. As shown in this study, the enzyme α-amylase may join this group of novel targets and may become another candidate affecting regulation of cell growth and providing new insights in proliferation control. In previous investigations of gene expression in mammary gland tissue from different rat strains, we unexpectedly discovered that salivary α-amylase might have an impact on cell proliferation [4,5]. This prompted us to review known facts about this enzyme and to perform for the first time experiments to elucidate its effects on proliferation in the breast tissue.

α-Amylases, a family of glycoside hydrolases mainly produced in the salivary glands and pancreas, play a well-known role in the metabolism of starch cleavage by scission on 1,4-α-glycosidic bonds [6]. In mammals, there are mainly two different genes AMY1 and AMY2 including occurrence of several haplotypes that encode salivary (type 1) and pancreatic (type 2) amylase, respectively [6]. α-Amylases are used as markers for clinical diagnosis of diseases, e.g. inflammation and tumors [7-9], exhibit antibacterial effects [10,11], and have been detected in the mammary gland [12], breast milk [13], vaginal secret [14], and many other tissues [15], but the function there is mostly unknown. α-Amylase has also been determined in lung tumors [16,17] and in a rare type of breast tumors [18,19]. The expression of the different α-amylases is tissue-specific; salivary α-amylase is the predominant α-amylase in the mammary gland [12]. Heitlinger et al. [13] suggested that α-amylase type 1 in the breast milk compensates for low salivary and pancreatic activity in newborns by improving energy utilization of solid nutrition.

Interestingly, there exist some hints for antiproliferative effects of α-amylase with unknown mechanism. At the beginning of the last century, Beard [20] used extracts of α-amylase type 2 and other pancreatic enzymes to treat patients with tumors in various tissues. Novak and Trnka [21] reported prolonged survival in amylase-treated mice after subcutaneous transplantation of melanoma cells. In comparisons of mouse strains with differing spontaneous mammary tumor incidence, blood α-amylase was positively correlated with tumor potential [22]. Malignant types of breast cysts in human patients contained lower α-amylase levels than cysts with widely benign behavior [23].

Among several factors, stress is one parameter that seems to promote breast cancer [24]. Salivary α-amylase has been recently introduced as an appropriate parameter for stress in humans that increases rapidly during stressful situations [25] reflecting the activity of the sympathoadrenergic system [26,27]. However, to our knowledge, no investigations on α-amylase levels or actions regarding mammary carcinogenesis have been published.

The objective of the present study was to examine if salivary α-amylase is able to alter growth of mammary epithelial cells by using primary cultures of rat origin. For this purpose, we used primary mammary epithelial cells from two inbred rat strains, Fischer 344 (F344) and Lewis, which originate from the same genetic background, the Sprague-Dawley outbred rat [28], but differ in their response to stress and sensitivity to carcinogens [29-31]. Moreover, we performed experiments with primary cultures from human breast tumors in order to compare α-amylase effects on different mammary cells from various sources and species. These investigations were expected to provide evidence if α-amylase serves as a new candidate for breast cancer prophylaxis or therapy.

## Materials and methods

### Animals

Female rats from two inbred rat strains, F344 and Lewis, were obtained from Charles River (Sulzfeld, Germany) at an age of about six weeks (42-45 days). In total, 18 F344 and 16 Lewis rats were used for five preparations per strain. Rats were housed in groups of 4-5 animals per cage with controlled conditions of temperature (23-24°C), humidity (about 50%), and light (12 h dark/light cycle; light off 6 p.m.). The experimental protocol was in line with national and international ethical guidelines, conducted in compliance with the German Animal Welfare Act, and approved by the responsible governmental agency, including approval by an animal ethics committee. All efforts were made to minimize pain or discomfort of the animals.

### Human cells

Primary human breast cancer-derived epithelial cells (HBCEC) from mammary carcinoma excisions were used to study the effect of salivary α-amylase in different mammary cells of human origin. Detailed information about derivation or source of these cells and their maintenance was described previously [32].

### Cell preparation and culture

Rats were killed at an age of 7-9 weeks by CO_2_-anesthesia and cervical dislocation for dissection of three paired mammary gland complexes (cranial cervical; abdominal; cranial inguinal). Cell preparation of the rat mammary glands was done according to the protocol of Bissell´s group for mouse tissue [33] in a modified way. Prior to dissection of mammary gland complexes, skin and fur were cleaned with ethanol (70%) or Braunol^® ^(Braun, Melsungen, Germany). Cells from about 20% of the animals, cleaned with ethanol, turned out to be infected mostly with fungi. The number of culture infections decreased from 20% to about 5% by use of the iodine-based disinfectant Braunol^®^. The mammary gland complexes were taken under sterile conditions and stored in ice-cold phosphate-buffered saline (PBS). For cell extraction, tissue was minced by scalpels and incubated in a pre-warmed enzymatic solution (0.2% trypsin, 0.2% collagenase A, 5% fetal calf serum, and 5 µg/ml gentamicin in Dulbecco´s Modified Eagle Medium with nutrient mixture F12 (DMEM/F12)) on a shaker for 70-90 min at 37°C. After centrifugation (1,500 rpm, 10 min), DNAse (40-50 U) was used for further cell dissociation (2-5 min, room temperature, manual shaking). Groups of epithelial cells were separated by pulse centrifugations from single cells that were supposed to be mainly fibroblasts. Epitheloids were seeded on plates (28 cm^2^, Cellstar, Greiner BioOne, Frickenhausen, Germany; one plate per animal) coated with Matrigel^® ^(BD Biosciences, Bedford, MA). Matrigel^® ^dilution was ten- or twelvefold in DMEM/F12. For cell culture, the Mammary Epithelial Cell Growth Medium (PromoCell, Heidelberg, Germany) with the supplement kit (bovine pituitary extract, human epithelial growth factor, bovine insulin, and hydrocortisone) was used. The antibiotics penicillin/streptomycin (100 U/ml and 100 µg/ml, respectively) and gentamicin (50 µg/ml) were added.

In contrast to the enzymatic digestion of rat mammary glands, HBCECs were obtained from explant cultures of human mammary tumor tissue. HBCECs and normal HMECs, as well as the primary rat mammary cells were cultured in an incubator at 37°C with 5% CO_2_, 95% fresh air and saturated humidity as described previously [32]. Change of medium was performed the day after preparation and then every two or three days.

These conditions for preparation and culture were successful in predominantly culturing mammary cells with an epithelial phenotype and to avoid a significant contamination with stromal cells, e.g. fibroblasts. Moreover, incubation with trypsin/ethylenediaminetetraacetic acid (EDTA) for 2-3 minutes at room temperature further eliminated fibroblasts due to different sensitivities of epithelial cells and fibroblasts towards trypsin.

For cell counting and passaging, trypsin/EDTA (0.15%) was used to detach cells, and its reaction was stopped with fetal calf serum (20%) in DMEM/F12. Remaining passage 0 (P0)-cells were allowed to proliferate again, so that a second seeding was possible.

Cell counting was performed within the Fuchs-Rosenthal-chamber. Cell viability was accessed by trypan blue exclusion (trypan blue final concentration 0.08%; Sigma, Schnelldorf, Germany).

Firstly, cells from mammary gland complexes of different locations were cultured separately. There were no obvious differences in morphology, behavior in culture, cell growth, and contamination with stromal cells, so that cells from all the excised mammary gland complexes per single animal were cultured together.

### Identification of epithelial and mesenchymal cells by immunocytochemistry

The proportion of epithelial cells in culture was determined by cytokeratin as epithelial cell marker. Additionally, expression of vimentin was determined, which is expressed in fibroblasts and mesenchymal precursor cells [34] but may also appear in cultured epithelial cells [35]. To distinguish between different populations of cells, double labeling of cellular cytokeratin and vimentin was performed. Cells were seeded on Matrigel^®^-coated cover slides in 24-well-plates. Fixation with methanol/acetone (1:1) was followed by washing with PBS, incubation with blocking solution (PBS with 1% bovine serum albumin and 0.25% Triton X), incubation with the first primary antibody (1 h, 37°C, monoclonal anti-pan-cytokeratin (clone PCK-26) from mouse, dilution 1:100; Sigma, Schnelldorf, Germany), washing, and incubation with Cy2-fluorescent-marked secondary antibody (30 min, 37°C, goat-anti-mouse, dilution 1:100, Jackson Immunoresearch, Dianova, Hamburg, Germany). After washing, monoclonal anti-vimentin antibody from mouse was added (1 h, 37°C, Cy3-labeled, dilution 1:200; Sigma, Schnelldorf, Germany). Finally, cell nuclei were stained with 4,6-diamidin-2-phenylindol (DAPI). All primary and secondary antibodies were diluted in blocking solution.

The proportions of cytokeratin- and vimentin-positive as a fraction of all DAPI-stained cells were evaluated microscopically (Zeiss Axioskop; Carl Zeiss Microimaging GmbH, Göttingen, Germany). Exclusively vimentin-positive cells were considered as fibroblasts, cytokeratin-positive or vimentin- and cytokeratin-positive cells were counted as epithelial cells.

### Detection of cellular α-amylase by immunocytochemistry

Visualization of α-amylase was performed by a primary anti-antibody against human salivary α-amylase (1 h, 37°C, fractionated antiserum from rabbit; dilution 1:50; Sigma, Schnelldorf, Germany), the secondary swine-anti-rabbit-antibody (30 min, 37°C, biotilinated; dilution 1:50; Dako, Hamburg, Germany), and Cy3-labeled-streptavidin (1 h, 37°C, dilution 1:1,000; Jackson Immunoresearch, Dianova, Hamburg, Germany). Nuclei were stained by DAPI. Determination of intracellular localization of α-amylase was done by confocal microscopy (Leica TCS SP5 II with AOBS (acousto optical beam splitter), Leica Microsystems, Wetzlar, Germany).

### α-Amylase treatment in rat cells

Salivary α-amylase (α-amylase from human saliva; 300-1,500 U/mg protein; Sigma, Schnelldorf, Germany) dissolved in sterile water was used for treatment *in vitro*. The batches of α-amylase used in the experiments contained a specific activity of 66.3 U/mg solid, which was considered for enzyme solvent preparation. The specific cells from all animals were merged, seeded onto 12-well- or 24-well-plates with a seeding density of 15,000 cells/cm^2 ^(seeding density in some experiments 12,000-20,000 cells/cm^2^), and cultured for 2-4 days (in one experiment 7 days) prior to α-amylase treatment. Finally, cells were detached with trypsin/EDTA, counted in a Fuchs-Rosenthal-chamber, and viable cells were determined by trypan blue exclusion. Evaluated data are shown as cells/well or as change in cell number compared to control treated wells in percentage.

α-Amylase concentrations for treatment of cells were not available from literature. Novak & Trnka [21] used α-amylase for *in vivo *treatment of mice with subcutaneous tumors (6-7 U/mouse in 0.1 ml). In order to define appropriate α-amylase concentrations for cell culture treatment, experiments were conducted with five different α-amylase concentrations (0.1 U/ml, 1, 5, 10, and 50 U/ml) applied to F344 and Lewis cells once per day for two days. In another experiment, different durations of α-amylase treatment (one day, two and four days) were performed in order to find proper conditions to examine α-amylase effects. In all following experiments, α-amylase (5 and 50 U/ml) was added once per day for two days to the wells after change of medium. Control cells were treated with vehicle (water). In the majority of experiments, cells derived from prepared P0-cells were treated with α-amylase (P1-cells).

As already mentioned, remaining P0-cells were further cultivated after a first seeding and could be harvested a second time (second seeding). All these cells were called P1-cells.

About half of the independently performed experiments (3 out of 7 for F344; 3 out of 6 for Lewis) were done in a blind fashion, meaning that the experimenter, who did the treatment and cell counting, was not aware about the treatment groups. In the first set of experiments, the experimenter knew about the treatment groups to be able to notice cellular alterations during α-amylase treatment. Experiments were evaluated individually and could be analyzed together because no differences were observed between blind- and non-blind-performed investigations.

### α-Amylase treatment in human mammary epithelial cells

The effect of α-amylase in mammary cells of human origin was studied in primary HBCEC (mammary carcinoma excisions). α-Amylase treatment was performed once per day for 2 days with 0.125 U/ml, 1.25 U/ml, 12.5 U/ml, and 125 U/ml. Control cells were treated with water.

### SA-β-galactosidase assay

Expression of senescence-associated-β-galactosidase (SA-β-gal) is increased in senescent cells [36]. To determine if α-amylase treatment causes a change in cell senescence, primary rat mammary cells were cultured on Matrigel^®^-coated 24-well-plates. Treatment with salivary α-amylase (5 and 50 U/ml) for 2 days started after 1 (P1) or 4 (P2) days in culture. The cells were fixed with 1x Fixative Solution, containing 20% formaldehyde and 2% glutaraldehyde and stained against SA-β-gal for 24 h/37°C in the dark according to the manufacturers protocol and recommendations (Senescence SA-β-galactosidase Staining Kit, Cell Signaling Technology, New England Biolabs, Frankfurt, Germany). The staining was proportional to the amount of substrate (5-bromo-4-chloro-3-indolyl-beta-D-galactopyranoside) enzymatically transformed. Following two washes with PBS, the differentially-stained cell cultures were documented by phase contrast microscopy using Olympus imaging software cell^® ^(Olympus, Hamburg, Germany) and quantified by counting.

Cells from F344 (P1 and P2) and Lewis (only P2) were counted in three different wells and portion of SA-β-gal-positive cells was determined (one well). Positive and negative cells were counted in 6-9 sections. Data are shown as percentage SA-β-gal-positive cells. Total cell numbers per group of 759-963 cells for P1 and 510-803 cells for P2 were counted. In addition to this, cells from a human breast tumor (MaCa 700) were also treated with α-amylase (0.125, 1.25, 12.5, and 125 U/ml) and used for a SA-β-gal assay (three sections per treatment). Total cell numbers of 266-691 cells were counted.

### Statistical evaluation of data

Data are mainly shown as change in number of cells (α-amylase-treated) compared to control treated cells in percent (mean and standard error of the mean (SEM)). The conversion to percentage was necessary to compare and merge experiments because absolute numbers varied naturally between experiments with different seeding densities. Statistical analysis was performed by One-way-ANOVA and the Bonferroni test for selected pairs or Two-way-ANOVA and Bonferroni test. A p-value of <0.05 was considered as significant difference.

## Results

### Primary mammary epithelial cells from female F344 and Lewis rats

**Figure 1:**
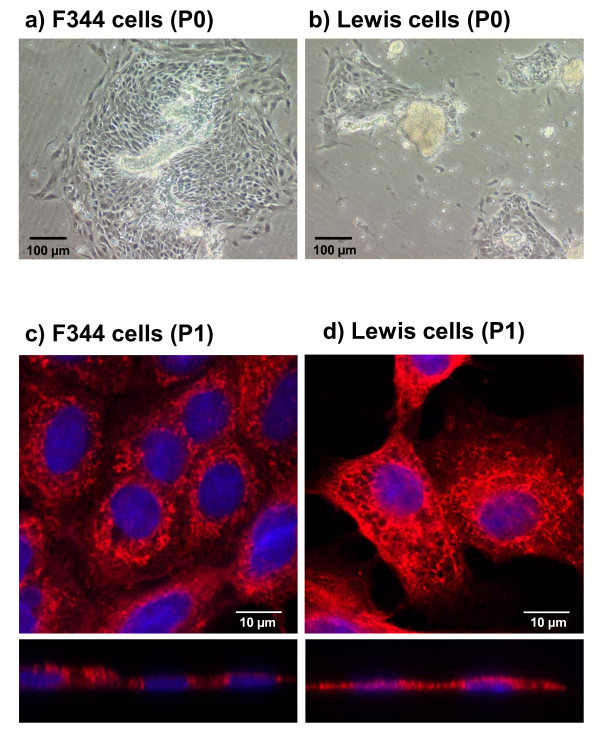
**Differences in cultures of primary mammary cells from F344 and Lewis rats and cellular localization of α-amylase**. One day after preparation, epitheloids from F344 (a) showed a faster and better attachment and a more effective growth in comparison to those from Lewis rats (b). Detection of α-amylase (Cy3; red) was performed in mammary gland cells from F344 (c) and Lewis (d) rats (P1). Nuclei were stained with DAPI (blue). Pictures show cells in xy- and xz-axis by confocal microscopy. α-Amylase was present in F344 and Lewis cells. However, in Lewis cells, α-amylase was distributed throughout the whole cell, whereas in F344 cells it was found in a more granular manner near the nuclei (xz-axis).

Preparation of the dissected mammary gland complexes produced comparable amounts of epithelial cells in F344 and Lewis rats. Marked differences between cells from F344 and Lewis rats could be observed one day after preparation. Whereas F344 cells attached easily onto the plates and immediately started to grow (Figure 1a), attachment and growth of Lewis cells did not show that progress (Figure 1b). Moreover, cells derived from Lewis showed signs of senescence (no growth, enlarged cell body) more quickly during culture than F344 cells.

### Immunocytochemical discrimination between epithelial cells and fibroblasts

As the tissue preparation and culture conditions were optimized for epithelial cells, the cell cultures predominantly comprised mammary epithelial cells. This was additionally determined by immunofluorescence analysis using cytokeratin as a marker protein. The mean proportion of cytokeratin-positive cells in five different preparations was about 94%, 46% of all cells were both, cytokeratin- and vimentin-positive. It is known that epithelial cells in culture might express vimentin [34], so that only those cells exclusively stained for vimentin were considered as mesenchymal cells (about 6%). There were no obvious differences in the cell fractions between F344 and Lewis cells (P1).

### Immunocytochemical detection of salivary α-amylase in F344 and Lewis cells

Salivary α-amylase was similarly expressed in cultured rat mammary epithelial cells from F344 and Lewis, showing its localization in the cytoplasm (Figure 1c,d). In F344 cells, however, α-amylase was associated closer to the nucleus in a more granular manner (Figure 1c), but was spread net-like throughout the whole cell body in Lewis cells (Figure 1d).

### Effects of α-amylase on cell growth in cells from F344 and Lewis rats

**Figure 2:**
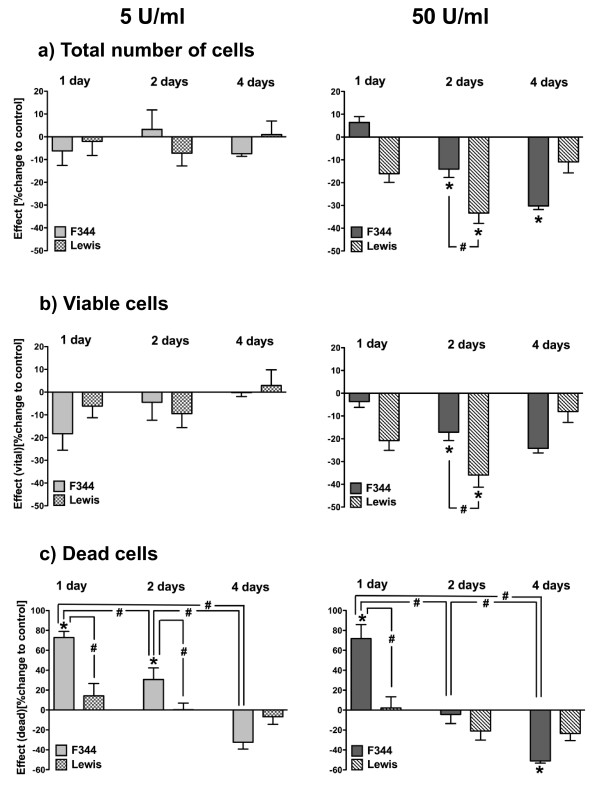
**Change in cell number after treatment of F344 and Lewis cells with salivary α-amylase for different incubation times**. The mean α-amylase effect is shown in percent as change compared to control cells treated with water for the total number of cells, exclusively viable, and for dead cells after 5 and 50 U/ml for 1 day, 2 days, and 4 days (n = 4-14 wells per group). For counting, cells were detached with trypsin/EDTA, and viable and dead cells could be determined by trypan-blue-exclusion. Results for total cell number and viable cells were comparable: there were no obvious differences after 5 U/ml α-amylase, but for 50 U/ml, a significant decrease in cell number was apparent after 2 days and more prominent in Lewis cells (a & b). Number of dead cells from Lewis rats was not influenced by amylase treatment (c). In contrast to this, dead cells from F344 rats markedly changed with duration of treatment in a similar way for 5 and 50 U/ml. After 1 day of α-amylase, the number was significantly increased, unchanged after 2 days, and significantly decreased after 4 days. Significant differences between controls and α-amylase are indicated by asterisk (p < 0.05); significant differences between treatment durations and F344 vs. Lewis are indicated by rhomb (p < 0.05).

It has not yet been described, if α-amylase has effects on mammary gland cell growth and, if, to what extent. Experiments with different α-amylase concentrations identified 5 and 50 U/ml as proper concentrations to reveal differences in α-amylase efficacy (not illustrated). In order to find the appropriate treatment duration, experiments were performed with α-amylase (5 and 50 U/ml) for one day, two, and four days (n = 4-14; Figure 2a). Cell numbers were not altered in F344 and Lewis cells after 5 U/ml for all treatments. After 50 U/ml, a significant decrease in number of cells was observed for Lewis cells after 2 days and also for F344 cells after 2 and 4 days (Figure 2a).

These results were evaluated from the total number of counted cells including viable as well as dead cells after detachment by trypsin. Comparable results were achieved when numbers of viable cells were evaluated (Figure 2b). In contrast, the number of dead F344 cells varied, depending on the duration of treatment but not on the α-amylase concentration (Figure 2c), whereas for Lewis, the amount of dead cells was not influenced by α-amylase (Figure 2c). Thus, prolonged α-amylase treatment reduced the number of non-viable cells in F344 cells, but not in Lewis cells.

**Figure 3:**
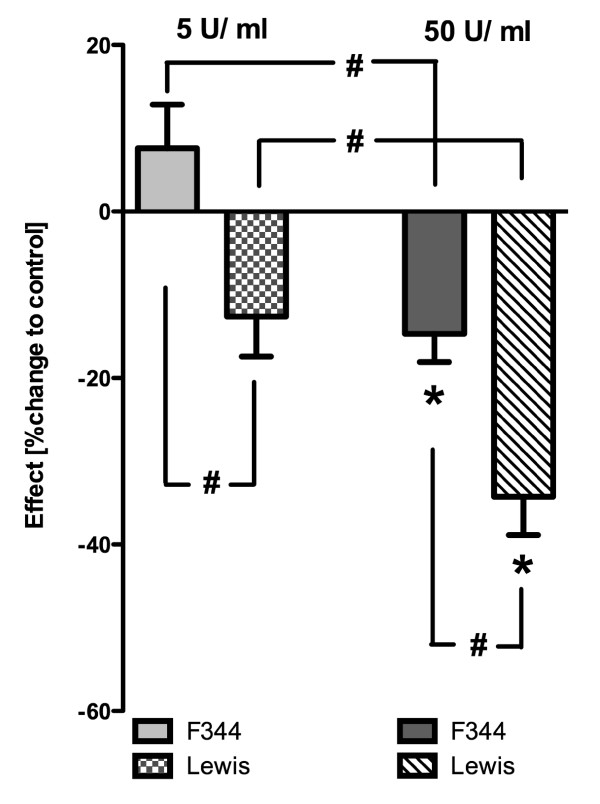
**α-Amylase effects on cell growth in F344 and Lewis cells after treatment for 2 days with 5 and 50 U/ml**. The mean α-amylase effect is shown as change in total cell number compared to the water-treated control cells (percent change; mean and SEM). Results from four to five different experiments were summarized and evaluated together for F344 and Lewis cells (n = 29-35 wells per group). Numbers of cells were significantly decreased after α-amylase treatment (50 U/ml) indicating antiproliferative effects. Lewis cells were significantly more sensitive towards α-amylase than F344 following incubation with both 5 U/ml and 50 U/ml. Statistics: One-way-ANOVA and Bonferroni for selected pairs: significant differences between controls and α-amylase are indicated by asterisk (p < 0.05); Two-way-ANOVA and Bonferroni: significant differences between F344 vs. Lewis and 5 U/ml vs. 50 U/ml are indicated by rhomb (p < 0.05).

Based on these experiments, the cells were treated with 5 and 50 U/ml α-amylase for 2 days (Figure 3). α-Amylase treatment with 50 U/ml significantly reduced the total cell number in F344 and Lewis cells indicating an inhibited cell proliferation. No significant alterations were seen after 5 U/ml compared to water-treated control cells. F344 cells showed significantly less sensitivity towards α-amylase in comparison to cells from Lewis rats after both concentrations (5 U/ml: +7.6% and -12.6%; 50 U/ml: -14.7% and -34.3% for F344 and Lewis, respectively; p < 0.05; Figure 3). The decrease in total cell number was concentration-dependent for cells from both rat strains (50 U/ml > 5 U/ml; p < 0.05).

### α-Amylase effects in mammary tumor cells of human origin

**Figure 4:**
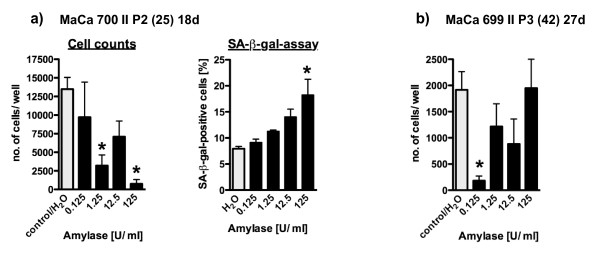
**Determinations of α-amylase effects in different cells of human origin**. For two HBCEC cultures, a significantly reduced cell number after α-amylase treatment was demonstrated (n = 2-6; mean and SEM). MaCa 700 responded in a dose-dependent manner (a). Additionally, the SA-β-gal assay was performed in MaCa 700 cells, and the proportion of SA-β-gal-positive cells was significantly increased by 125 U/ml α-amylase. The latter parameter showed a tendency for concentration-dependency (Pearson´s correlation coefficient 0.9002; not significant). In MaCa 699 cells, only the lowest concentration caused a significantly decreased cell number (b). Asteriks indicate significant differences vs. control cells (One-way-ANOVA and Bonferroni for selected pairs, p < 0.05).

Mammary cells from human breast tumors were also treated with α-amylase for two days. Similar to differences between F344 and Lewis cells, sensitivity towards salivary α-amylase differed depending on the origin (or source) of the cells. Cells from two different human breast tumor patients were treated with four different concentrations of α-amylase (0.125, 1.25, 12.5, and 125 U/ml). Statistical analysis revealed that cells cultured from one tumor (mammary carcinoma (MaCa) 700 II P2; Figure 4a) showed significant decreases in cell number after 1.25 and 125 U/ml (-76% and -94.6%). Cells from the other tumor (MaCa 699 II P3; Figure 4b) only significantly responded to the lowest concentration (0.125 U/ml: -90.5%).

Primary cells from another human breast tumor that had been cultured for 296 days did not respond with a change in cell number. In contrast, a culture of an invasive ductal human breast tumor showed a concentration-dependent decrease in number of cells in comparison to water-treated control cells. Results from these cells were not statistically analyzed because only one well per treatment was done.

### Cell senescence after α-amylase treatment

A possible influence of α-amylase on cell senescence was investigated by determination of SA-β-gal-positive cells. Without treatment, P2-F344 cells showed significantly increased numbers of SA-β-gal-positive cells compared to P1-cells (2-3fold). There were no significant differences in cell growth or SA-β-gal-positive cells after 5 U/ml. α-Amylase at 50 U/ml significantly decreased number of cells in P1-F344 cells, but not in P2-F344 or P2-Lewis, although there was a tendency for P2-F344 (Table 1). Alteration in SA-β-gal-positive cells was not strictly combined with a change in cell number after α-amylase, because cell counts were decreased in P1-F344 cells, but SA-β-gal-positive cells were not changed. Moreover, there was a significant increase in SA-β-gal-positive P2-F344 cells by 50 U/ml, but no significant alteration in number of cells (Table 1). Lewis cells (P2) did not respond to α-amylase in this experiment.

**Table 1:** SA-β-gal assay and cell number after α-amylase treatment in F344 and Lewis cells

	F344, P1	F344, P2	Lewis, P2

**SA-β-gal assay**	SA-β-gal-positive cells (%)	SA-β-gal-positive cells (%)	SA-β-gal-positive cells (%)

**Control (H_2_O)**	11.94 ± 1.81	27.35 ± 3.28	33.82 ± 1.48

**5 U/ml α-amylase**	13.86 ± 1.41	37.15 ± 3.19	34.12 ± 3.20

**50 U/ml α-amylase**	11.83 ± 2.39	39.48 ± 3.47*	29.81 ± 2.78

	n.s.	*H_2_O vs. 50 U/ml	n.s.

	**F344, P1**	**F344, P2**	**Lewis, P2**

**Cell counts**	Number of cells/well	Number of cells/well	Number of cells/well

**Control (H_2_O)**	17,250 ± 1,377	4,500 ± 577	4,188 ± 567

**5 U/ml α-amylase**	17,958 ± 1,514	3,958 ± 240	5,292 ± 163

**50 U/ml α-amylase**	11,833 ± 870*	2,371 ± 344	4,483 ± 464

	*H_2_O vs. 50 U/ml	n.s.	n.s.

α-Amylase (50 U/ml) decreased the number of cells only in P1-F344-cells, but not in P2-F344- and P2-Lewis-cells. Proportion of SA-β-gal-positive cells did not correlate with cell number, as this amount of cells was not altered in P1-F344 cells, but significantly increased in P2-F344 cells after 50 U/ml α-amylase. No difference at all was observed in Lewis-cells (P2) and after 5 U/ml α-amylase. Mean and SEM are shown for three wells per group (cell counts) or 6-9 sections (SA-β-gal assay). Significant differences (p < 0.05) vs. control cells (One-way-ANOVA and Bonferroni for selected pairs) are indicated by asterisk.

In MaCa 700 cells, a primary culture from a human breast tumor, α-amylase caused a significant decrease in number of cells after 1.25 and 125 U/ml α-amylase for 2 days (Figure 4a). The portion of SA-β-gal-positive cells was significantly increased only after 125 U/ml. However, there was a tendency for a concentration-dependent increase of SA-β-gal-positive MaCa 700 cells (Figure 4a).

## Discussion

The experiments described here revealed for the first time that salivary α-amylase exhibits *in vitro *antiproliferative effects in primary rat mammary epithelial cells and human breast tumor cells. On the one hand the effects on healthy rat breast cells indicate that endogenous α-amylase might be involved in the regulation of mammary cell proliferation, and on the other hand the results of human breast tumor cells suggest that it might provide a useful tool for tumor prophylaxis or therapy. α-Amylase concentrations and treatment duration were determined experimentally because to our knowledge only one previous experimental study exists that used α-amylase for tumor treatment. In this study, Novak & Trnka [21] found prolonged survival in mice with transplanted B16F10 cell melanoma after subcutaneous application of α-amylase. In the latter study, pancreatic α-amylase was used to follow the protocol of Beard [20], who used crude pancreas extract. However, effects of salivary α-amylase on cell growth *in vitro *as described here have not been previously reported in the literature. The present experiments were performed with salivary α-amylase, because the mammary and the salivary glands share certain similarities in their embryology [37], and salivary amylase is the isoenzyme present in the breast milk [38]. Although it remains unclear if pancreatic α-amylase exhibits similar effects on cell growth, previous work has reported that both isoenzymes vary in their activities on distinct substrates [39,40] suggesting different properties on mammary cell proliferation.

Interestingly, sensitivity towards α-amylase varied depending on the cell origin. Mammary cells from Lewis rats were quite sensitive and showed stronger effects compared to F344 rats. Cells from human breast tumors also responded in different ways showing distinct sensitivity. Thus, the impact of α-amylase on cell growth *in vitro *depends on cellular conditions, origin, e.g. rat strain, and distinct cellular characteristics.

The rat primary cells in this study were derived from F344 and Lewis rats that are histocompatible inbred rat strains originating from the same background strain [28], but with differing responses towards stress [30,41], indicating a stronger stress response of F344 compared to Lewis rats. Determination of α-amylase was not performed in these studies.

In line with the diverse stress response, F344 rats show a higher tumor incidence compared to Lewis, particularly after exposure to many known carcinogens, which is attributed to the higher levels of immunosuppressive cortisol in F344 [29]. On the other hand, Lewis appear to be more susceptible to autoimmune diseases according to the low cortisol values, which were observed in this rat strain [29]. Previous investigations from our group showed that cell proliferation in mammary gland tissue was significantly increased in F344 rats, and not in Lewis, after magnetic field exposure [42], which is considered to act as a stressor to sensitive tissues [43-45].

Just a few years ago, salivary α-amylase was discovered as a stress parameter in humans that, in contrast to cortisol, reflects the sympathetic-adrenergic activity [27] and rapidly increases by stimulation of β-adrenergic receptors [26]. Due to low α-amylase sensitivity, stress influences might cause a less regulated cell proliferation in F344 breast tissue. In contrast to this, mammary Lewis cell proliferation was well regulated showing rather soon signs of senescence. These considerations are supported by the observation that F344 cells attached easier and grew faster than Lewis cells (Figure 1a & b). α-Amylase was detected in both, F344 and Lewis primary mammary epithelial cells (Figure 1c & d) without obvious differences. Moreover, we recently determined amylase enzyme activity in the mammary gland tissue of F344 and Lewis rats and observed no differences in activity between both rat strains (unpublished data). These findings indicate that other factors than α-amylase protein expression and activity must underlie the observed differences. Thus, the α-amylase efficacy on its targets is probably altered in F344 cells participating in less regulation of cellular proliferation.

However, the enzymatic preparation of mammary gland tissue might alter cell surface and therefore influence adhesion properties *in vitro*. Microenvironmental influences in the breast tissue, which strongly affect cellular behavior [46-48] and which are absent or at least altered in our primary cultures *in vitro*, should also be considered.

Currently, the possible mechanisms underlying antiproliferative effects of α-amylase remain unclear. However, some sources in literature can be found that allow considerations about a possible mechanism and probable α-amylase targets. α-Amylase might act on molecules, which mediate cell adhesion, and stimulate detachment and death of cells called anoikis, a type of apoptosis [49,50]. In our experiments, the proportion of dead cells reflects the sensitivity to trypsin used for cell detachment prior to counting. If α-amylase induces anoikis by action on cellular adhesion, a more pronounced trypsin effect would have been expected that is negatively correlated with number of cells. This was not the case in either, F344 and Lewis cells.

Furthermore, α-amylase could probably stimulate cellular differentiation or senescence. Investigations of cell senescence by SA-β-gal assay presented here did not show a strong impact of α-amylase on senescence, particularly not in combination with the effect on cell growth.

α-Amylase also exerts antibacterial effects, which are either drawn back to an inhibition of bacteria growth by diminishing nutrients [10] or to a direct interaction with α-amylase [11]. Regarding cell culture, known α-amylase-substrates, like starch, are usually not present in cell culture media, but an α-amylase effect by metabolism of nutrients cannot be completely excluded. F344 and Lewis cells were cultured simultaneously with medium of the same composition, so that differing dependence on growth influencing substances could be a possible reason for the observed differences.

Another explanation for the α-amylase effect on cell growth might be an interference with growth stimulating hormones, e.g. estrogens. Hahnel et al. [51] showed *in vitro *that α-amylase inhibited or diminished binding of estradiol to its receptor. Previously, a correlation between α-amylase and hormone levels was reported *in vivo *[14], and hormonal alterations during sexual cycle influenced α-amylase activity in rat ovaries [52].

*In vivo*, the sympathetic system and its adrenergic receptors are activated during stress. α-Amylase is stimulated by adrenergic receptors [25] and probably adjusts or counteracts proliferation that has been elicited by α- and β-adrenergic receptors induced by stress. It is known that the mammary gland is innervated by sympathetic fibers. Mammary epithelial cells express α- and β-receptors, the receptor densities are hormone-dependent, and cell proliferation is influenced by these receptors [53-56], so that there might be a possible connection or interaction between estrogens, adrenergic receptors and α-amylase, which has not yet been described. In F344 cells, adrenergic receptors might stimulate proliferation in a more pronounced way due to intensive activation by stress that could not be effectively regulated. According to this hypothesis, cell proliferation in Lewis rats is affected by adrenergic receptors in a more moderate way and can easily be adjusted by α-amylase.

In summary, the present results demonstrate antiproliferative properties of salivary α-amylase in mammary epithelial and breast tumor cells suggesting that α-amylase might constitute a new strategy to prevent or treat breast cancer. However, the reasons for the altered cellular sensitivity towards α-amylase should be identified to allow a reliable prediction which type of breast cancer cells can be sufficiently inhibited in proliferation to ensure an appropriate efficiency of tumor treatment. The stimulation of endogenous α-amylase secretion and activity in the vicinity of the neoplastic tissue may provide a reasonable approach to affect tumor growth. Consequently, a direct administration of α-amylase into or nearby the tumor could represent a conceivable opportunity to monitor both, anti-tumor and potential side effects.

## Conclusions

To our knowledge, the findings presented here indicate for the first time that α-amylase plays a role in the regulation of mammary cell proliferation. However, the underlying mechanisms and the influencing factors of α-amylase's action must be further elucidated. In view of the potential impact on regulation of mammary cell proliferation, determination of α-amylase might be used to distinguish the risk for cancer development, and α-amylase may provide an interesting new target for tumor prophylaxis and treatment.

## Abbreviations

ACTH: adrenocorticotropic hormone; BSA: bovine serum albumin; Cy: cyanine dyes; DAPI: 4,6-diamidino-2-phenylindole; DMBA: 7,12-dimethylbenz[*a*]anthracene; DMEM: Dulbecco´s Modified Eagle Medium; EDTA: ethylenediaminetetraacetic acid; F12: nutrient mixture F12; F344: Fischer 344; HBCEC: human breast cancer-derived epithelial cells; L/R1: left/right mammary gland complex at cranial cervical location; MaCa: mammary carcinoma; P1: cell passage 1; PBS: phosphate-buffered saline; SA-β-gal: senescence-associated-β-galactosidase; SEM: standard error of the mean

## Competing interests

The authors declare that they have no competing interests.

## Authors' contributions

MF participated in the design of the study, primary rat mammary cell preparation and culturing, performed the cell counting, immunofluorescence staining and statistical analysis and drafted the manuscript. RH provided the human breast tumor cells and expert views in primary cell culture methods, participated in the SA-β-gal staining and helped draft the manuscript. CB performed experiments with the human cells and the SA-β-gal staining. WL participated in the design of the study and helped draft the manuscript. All authors read and approved the manuscript.
